# Evaluation of the Corrosion Resistance of Different Types of Orthodontic Fixed Retention Appliances: A Preliminary Laboratory Study

**DOI:** 10.3390/jfb14020081

**Published:** 2023-01-31

**Authors:** Busra Kumrular, Orhan Cicek, İlker Emin Dağ, Baris Avar, Hande Erener

**Affiliations:** 1 Department of Orthodontics, Faculty of Dentistry, Zonguldak Bulent Ecevit University, Zonguldak 67100, Turkey; 2 Department of Metallurgical and Materials Engineering, Faculty of Engineering, Zonguldak Bulent Ecevit University, Zonguldak 67100, Turkey; 3 Department of Orthodontics, Faculty of Dentistry, Tekirdag Namık Kemal University, Tekirdağ 59030, Turkey

**Keywords:** orthodontics, lingual retainer, electrochemical corrosion, pitting corrosion, current density, corrosion rate, polarization curve

## Abstract

(i) Objective: The present study aimed to compare the electrochemical corrosion resistance of six different types of fixed lingual retainer wires used as fixed retention appliances in an in vitro study. (ii) Methods: In the study, two different Ringer solutions, with pH 7 and pH 3.5, were used. Six groups were formed with five retainer wires in each group. In addition, 3-braided stainless steel, 6-braided stainless steel, Titanium Grade 1, Titanium Grade 5, Gold, and Dead Soft retainer wires were used. The corrosion current density (*i_corr_*), corrosion rate (CR), and polarization resistance (*R_p_*) were determined from the Tafel polarization curves. (iii) Results: The corrosion current density of the Gold retainer group was statistically higher than the other retainer groups in both solutions (*p* < 0.05). The corrosion rate of the Dead Soft retainer group was statistically higher than the other retainer groups in both solutions (*p* < 0.05). The polarization resistance of the Titanium Grade 5 retainer group was statistically higher than the other retainer groups in both solutions (*p* < 0.05). As a result of Scanning Electron Microscope (SEM) images, pitting corrosion was not observed in the Titanium Grade 1, Titanium Grade 5 and Gold retainer groups, while pitting corrosion was observed in the other groups. (iv) Conclusion: From a corrosion perspective, although the study needs to be evaluated in vivo, the Titanium Grade 5 retainer group included is in this in vitro study may be more suitable for clinical use due to its high electrochemical corrosion resistance and the lack of pitting corrosion observed in the SEM images.

## 1. Introduction

In orthodontic treatment, relapse is defined as the return of the teeth to their initial positions or their positions failure result of the treatment [1]. Riedel defined retention as: “Retaining the teeth in an ideal aesthetic and functional position” [2]. Retention, in orthodontics, is defined as the treatment that allows teeth to stay in their proper positions after the treatment is finished and creates the last stage of orthodontic treatment [3]. The appliances used in retention are divided into two groups: removable and fixed. Fixed retention appliances are often preferred because they do not require the patient’s cooperation. In addition, they are aesthetic due to their adhesion to the lingual surfaces of the tooth and provide better retention than removable retention appliances [4]. Although there are different approaches applied to retention after orthodontic treatment, most orthodontists recommend lifetime retention [5,6].

Stainless steel, nickel-titanium, cobalt-chromium, beta-titanium, and multi-stranded wire metal alloys are frequently used in orthodontic treatment [7].

Corrosion is an electrochemical process that leads to the breakdown of metal [8]. The corrosion rate is defined as the amount of metal dissolved per unit of time and provides a numerical assessment of the corrosion resistance of materials [9].

Whilst the corrosion rate is determined by the mass reduction method in chemical events, it is evaluated by the linear polarization method, Tafel polarization method, harmonic analysis, dynamic electrochemical impedance, and electrochemical impedance in electrochemical events [9]. Corrosion can be assessed by obtaining the polarization curves in solution with the electrochemical measurements [9]. While the electrode potential is changed within a determined range in the potentiodynamic method, the current density corresponding to this potential is measured. It not only gives information about the corrosion rate, but also about the corrosion mechanism [9].

Electrochemical corrosion is possible in the oral environment because saliva is a weak electrolyte [10,11]. The electrochemical properties of saliva depend on the concentrations of its ingredients, pH, surface tension, and buffering capacity. Hence, the corrosion process can be controlled by these variables [12].

The corrosion resistance of orthodontic alloys is affected by the oral environment, with various variables, such as temperature, amount and quality of saliva, plaque, pH, proteins, and physical-chemical properties of food [13,14]. As the wires used in orthodontic treatment stay in the mouth for a long time, they should be corrosion resistant, prevent ion release, and not cause allergic reactions. In other words, orthodontic wires should be biologically compatible with oral tissues. The corrosion of orthodontic wires not only reduces the mechanical properties of the wire, but also increases the metal ion release in the wire [15,16]. It is stated that nickel, chromium, and iron, which can be released by the corrosion of orthodontic wires, are considerably harmful elements [17,18,19].

It has been reported that systemic disease may occur due to titanium [20]. Titanium may be the cause of ‘yellow nail syndrome’. In 30 patients with yellow nail syndrome, energy dispersive X-ray fluorescence (EDXRF) was used to measure the titanium content. In the patients’ nails, the titanium content was found to be high, and the cause of yellow nail syndrome was determined to be titanium. Yellow nail syndrome is characterized by nail changes, bronchial obstruction and lymphedema. Sinusitis, associated with postnasal drip and cough, were the most common symptoms in patients with yellow nail syndrome [21]. Due to corrosion and wear, the particles and ions of titanium and titanium alloy components can accumulate in the surrounding tissues and inflammatory reactions can occur [20].

In the literature, there are many studies on the electrochemical corrosion of archwires used in orthodontic treatment [22,23,24]. However, there are not enough studies on the electrochemical corrosion of the retainer wires used as fixed appliances in retentions that are intended to remain in the mouth longer than the applied orthodontic treatment period, or even for a lifetime.

The present study is aimed to compare and evaluate the electrochemical corrosion resistance of six different types of fixed lingual retainer wires used as fixed retention appliances, in vitro, in pH 7 and pH 3.5 Ringer solutions, by considering the current densities, corrosion rates, and polarization resistances.

## 2. Materials and Methods

The ethics committee approval was obtained from the Non-Interventional Clinical Research Ethics Committee of Zonguldak Bulent Ecevit University (Decision no: 2022/06-23/03/2022).

The sample size calculation was performed in the G*Power 3.1.9.7 program. The effect size was calculated by using the means and standard deviations of the groups. The a error probability was set to 0.05. The power of the study (1-α error prob) was set to 0.95. According to these data, the actual power of the study was calculated to be 95% and the total sample size should have been 12. In the study, 60 retainer wires sample, 5 in each group, were used. The groups in this study were formed by selecting six different types of retainer wires from two different brands. Each group consisted of five samples, given below:

 Group 1: 0.50 mm diameter 3-braided stainless-steel retainer (Dentaurum, Ispringen, Germany)
 Group 2: 0.45 mm diameter 6-braided stainless-steel retainer (Dentaurum, Ispringen, Germany)
 Group 3: 0.50 mm diameter three braided Titanium Grade 1 retainer (Dentaurum, Ispringen, Germany)
 Group 4: 0.50 mm diameter three braided Titanium Grade 5 retainer (Dentaurum, Ispringen, Germany)
 Group 5: 0.50 mm diameter three braided Gold retainer (Dentaurum, Ispringen, Germany)
 Group 6: 0.50 mm diameter Dead Soft Respond Wire retainer (Ormco, CA, USA)

**Figure 1:**
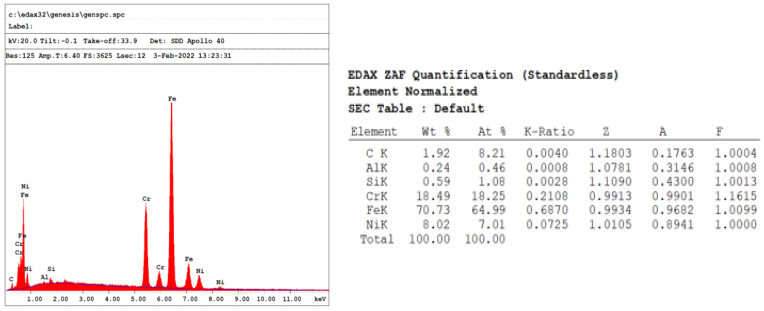
EDX analysis of Dead Soft retainer.

The equivalent weights and densities according to the ratios of the elements in the wires are given in Table 1 [25,26,27,28]. The Dentarum shared information about the chemical contents of the samples used in the study and the Ormco did not indicate it due to trade secrets. Hence, the chemical content of the Dead Soft retainer wire was determined using Energy Dispersive X-ray Analysis (EDX) [29] (Figure 1). The carbon content on the EDX analysis of Group 6 was ignored in the calculations; as no stainless steel includes the EDX method, one cannot truly analyze the amount of light elements it contains [30].

The surface area of the tested materials was adjusted to 0.239 cm^2^. The wires were coated with nail polish (Flormar, Italy), with the exception of the corroding portion, to prepare the samples for analysis. Each wire was ultrasonically cleaned with ethanol for 5 min before testing.

**Figure 2:**
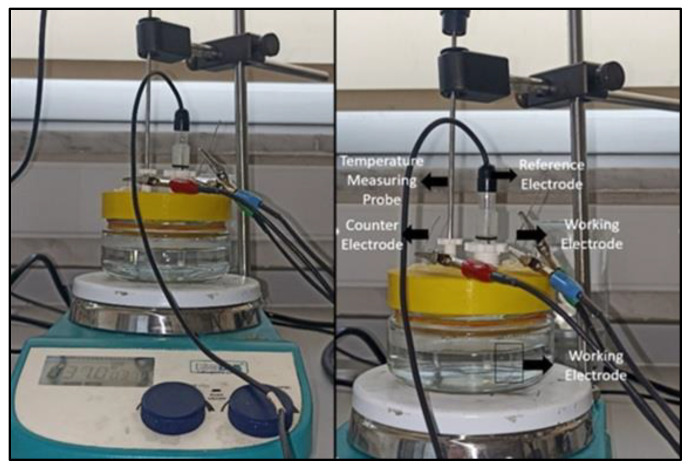
Three electrode system used for potentiodynamic polarization tests in Ringer’s solution at 37 ± 1 °C.

The Ringer’s solution consisted of 9 g/L Sodium Chloride (NaCl), 0.42 g/L Potassium Chloride (KCl), and 0.25 g/L Calcium Chloride (CaCl2). [31,32,33]. In order to adjust the pH of the solutions, 0.1 M Hydrogen Chloride (HCl) [34] and 0.1 M Sodium Hydroxide (NaOH) [35] were used to obtain pH 3.5 and pH 7 electrolytes. The corrosion cell was designed using the Solidworks 2014 computer aided design (CAD) program and was 3D printed from a 1.75 mm diameter thermoplastic polyurethane (TPU) filament. To prevent the formation of noise during the electrochemical testing, and to acquire reliable findings for every test, all of the experimental units were compactly aligned. As it can be seen in Figure 2, the potentiodynamic polarization tests were conducted at 37 ± 1 °C in the Ringer’s solution using a 3-electrode corrosion cell. Ag/AgCl was used as the reference electrode, platinum wire was conducted as the counter electrode and the retainer wire was applied for the working electrode.

After the test mechanism was set up, the temperature gradually increased until it reached 37 ± 1 °C. When the temperature became 37 ± 1 °C, the lingual retainer (working electrode) was kept in the solution for 1 h to provide an open circuit potential. The potentiodynamic polarization tests were conducted with a scan rate of 1 mV/s, from −1000 mV to +1000 mV, using the electrochemical workstation (Gamry Interface 1000E Potentiostat; Gamry Instruments Inc. 72 Warminster, PA, USA).

**Figure 3:**
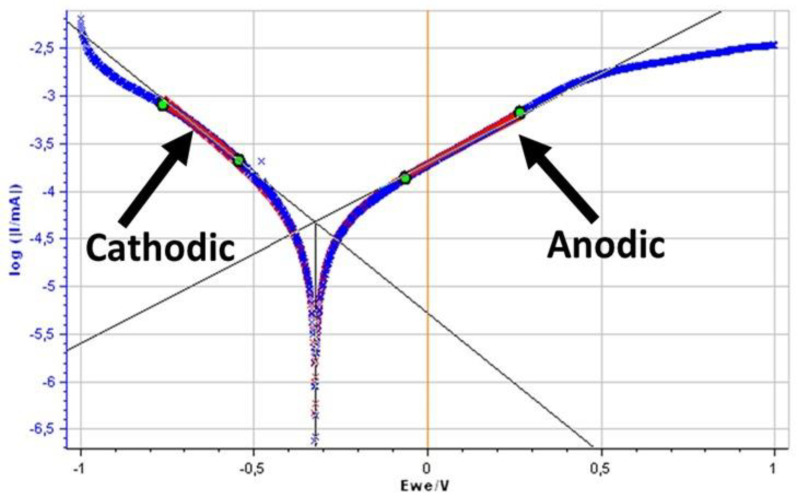
Test result of Ti6Al4V retainer wire-Test 3 and Tafel Extrapolation on EC-Lab Program.

The corrosion rate was determined using Tafel curves. The first thing to analyze using the Tafel curves is to determine the corrosion rate, which involves finding the corrosion current density; this can be calculated by drawing tangents to the anodic and cathodic tafel curves, then intersecting them, as shown in Figure 3 [36].

After finding the corrosion current density, The formula in the ASTM G 59 97 standard [36], given in (1), was applied to determine the corrosion rate of the retainer wires.

(1) $$CR=\frac{K1\times i_{corr}\times \mathrm{EW}}{\rho }$$

Here, in Formula (1), *i_corr_* indicates the corrosion current density (µA/cm^2^), EW is the equivalent weight of the material, K1 stands for the constant coefficient of 3.27 × 10^−3^ (mm·g/A·cm·year), ρ denotes the density (g/cm^3^), and *CR* defines the corrosion rate in (mm/year).

The polarization resistance (*R_p_*) (Ωcm^2^) was obtained using the Stern-Geary equation, shown in (2) [9,16,37].

(2) $$i_{corr}=\frac{1}{2303R_{p}}\left(\frac{b_{a}\times b_{c}}{b_{a}+b_{c}}\right)$$

Here, for the above formula, *i_corr_*, *R_p_* indicates the corrosion current density (A/cm^2^) and polarization resistance (ohms.cm^2^), while *b_a_, b_c_* denotes the anodic, cathodic Tafel slopes (volts/decade).

The samples’ surface morphology was evaluated through Scanning Electron Microscope (SEM) analysis.

The average, standard deviation, median, lowest, highest, frequency and ratio values were used in the descriptive statistics of the data. Kolmogorov-Smirnov tests were performed to determine whether the intra-group data were distributed. The Kruskal-Wallis test was used to see if there is a difference between the groups. The Mann-Whitney U test was used to find out which groups were different. In addition, the Mann-Whitney U test was used in the group comparison between the solutions. Statistical analysis was performed using the SPSS (version 28.0; SPSS, Chicago, IL, USA).

## 3. Results

**Figure 4:**
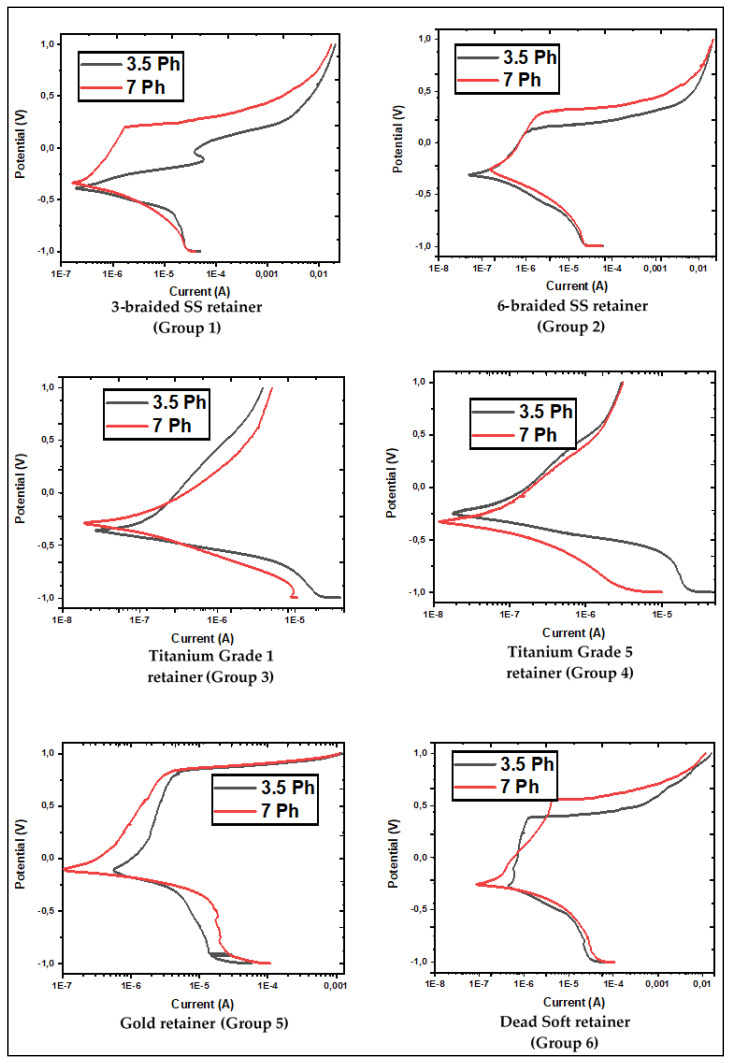
Electrochemical analysis. Red line: Mean potentiodynamic polarization curve in pH 7 Ringer’s solutions, Black line: Mean potentiodynamic polarization curves in pH 3.5 Ringer’s solutions.

The corrosion current density (*i_corr_* (µA/cm^2^)) is shown in Table 2; the corrosion rate (mm/year) and the polarization resistance (*R_p_* (Ωcm^2^)) test results are indicated in Table 3 and Table 4, respectively. The potentiodynamic polarization curves for all of the retainers and electrolytes are displayed in Figure 4.

### 3.1. Polarization Test Results

**Figure 5:**
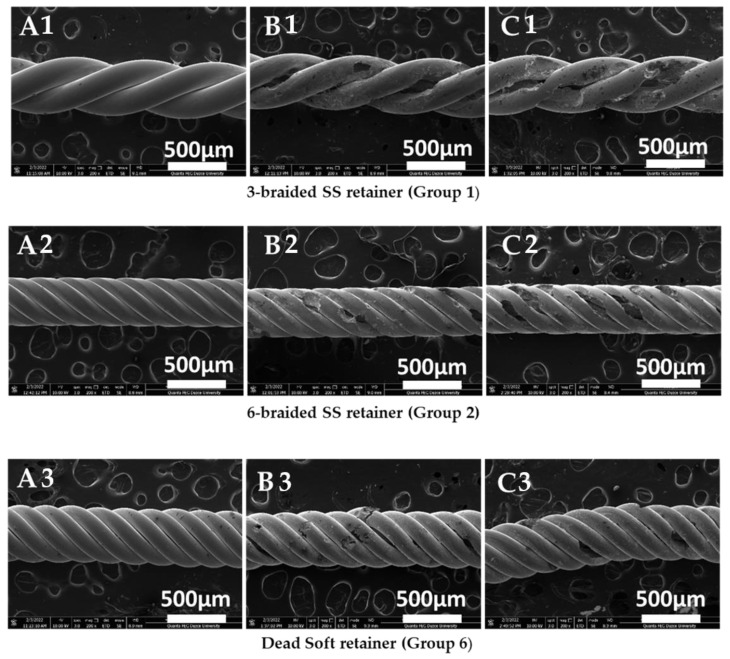
Scanning electron microscopy observations at 200× magnification. For Group 1: (**A1**); Before Experiment, (**B1**); pH 7 Ringer’s solution, (**C1**); pH 3.5 Ringer’s solution. For Group 2: (**A2**); Before Experiment, (**B2**); pH 7 Ringer’s solution, (**C2**); pH 3.5 Ringer’s solution. For Group 6: (**A3**); Before Experiment, (**B3**); pH 7 Ringer’s solution, (**C3**); pH 3.5 Ringer’s solution. (Scale bars for all groups: HV 10.00 kV, spot 3.0, mag 200×, det ETD, mode SE, WD 8.3-9.8 mm, 500µm, Quanta FEG).

The corrosion of metallic wires is an electrochemical phenomenon in which two reactions occur simultaneously in a conductive solution. The oral cavity is exposed to different pH by drinking and eating. The corrosion rate and type are affected by the kind of electrolyte, metal, production technique, test settings, and varying pH [8]. Table 2, Table 3 and Table 4 show that when the corrosion behavior of stainless steel (group 1, 2 and 6) retainer wires is evaluated, the corrosion rate increases as the pH drops. Among the stainless steel groups, the change in pH had the least effect on the 3-braided retainers. The corrosion current density for these wires, at 3.5 and 7 pH, had average values of 1.04 and 1.05 μA/cm^2^, respectively, as shown in Table 2. However, in the 6-braided Dentaurum and Deadsoft Respond wire retainers, the low pH increased the corrosion current density by approximately 144% and 79%. Diverse researchers have also investigated how pH impacts the electrochemical corrosion behavior of stainless steel orthodontic wires. Močnik et al. studied how the pH value of the solution effects the corrosion of NiTi and 304 stainless steel dental archwires. While the initial artificial saliva had a pH of 6.5, lactic acid was added to achieve 2.5 and 3.9 pH, and the corrosion current density values of NiTi and 304 steel were also compared. As the pH ratio decreased for the NiTi wires, the corrosion current density increased from 0.17 µA/cm^2^ to 0.83 µA/cm^2^. Similarly, the corrosion current density in stainless steel increased from 0.15 A/cm^2^ to 0.35 A/cm^2^ as the pH dropped [38]. In our study, the 6-braided Dentaurum SS had the highest corrosion resistance among the stainless steel retainers. The manufacturing differences between Deadsoft and Dentaurum, or the variation and inhomogeneities of the normalization annealing after production, may be responsible for the high corrosion resistance of the 6-braided wires, whose corrosion current densities are 0.27 and 0.66 μA/cm^2^ in 7 and 3.5 pH, respectively. Makiewicz et al. conducted the potentiodynamic polarization test on 304 stainless steel orthodontic archwires made by the 3M (USA) and Rocky Mountain Orthodontic [RMO] (USA) companies under the same test conditions and solutions. The corrosion current density for the RMO was 0.27 μA/cm^2^, whereas it was 0.49 μA/cm^2^ for the 3M [39]. The differences in the corrosion current density, corrosion rate, and polarization resistance between the two Dentaurum wires can be explained by the stresses caused by twisting while manufacturing, or by the localized corrosion, which affects the continuity of the passive Cr_2_O_3_ film formed on the surface of stainless steels. Furthermore, the difference in the heat treatments during and after wire production could have contributed to this. According to Zhang et al. different stress effects influence the corrosion rate and mechanism of stainless steel archwires [40]. Pitting corrosion may occur as a result of irregularities caused by production, the presence of salt containing chlorine ions, such as NaCl, KCl, or localized corrosion [41]. As can be seen in Figure 5, the pitting corrosion impacted all of the stainless steel groups. However, severe corrosion caused direct material loss in the 3.5 pH solution in the 3-braided Dentaurum wire. The main reason for the pitting corrosion being so effective is the aggressive ions in the Ringer’s solution. Titanium has excellent corrosion resistance due to the passive protective TiO_2_ film formed on the surface of titanium and its alloys [42]. The presence of corrosive ions, such as Cl^−^ in the electrolyte, may cause the corrosion of titanium and its alloys, as in stainless steel. As with stainless steel, the corrosion of titanium grade 1 and 5 accelerated as the pH decreased. The corrosion current density of the Ti-6Al-4V alloy was found to be 0.12 and 0.13 μA/cm^2^, while titanium grade 1 had 0.22 and 0.25 μA/cm^2^. Similarly, Calderón et al. reported the corrosion current density of Ti-6Al-4V to be 0.044 µA/cm^2^ and 0.07 µA/cm^2^ for pure Ti. For the phosphate buffered solution, the Ti-6Al-4V alloy showed higher corrosion resistance than the pure titanium [43]. The gold retainer had the highest corrosion current density in our experiments. Although pure gold exhibits very noble behavior and does not corrode, the high corrosion current density may be due to a microgalvanic effect that may occur between Cu, Ag, Pt elements and gold. In addition, the continuity of the gold layer may be absent. In addition, irregularities that may occur while bending the wires, depending on the production method, may cause local corrosion. High stresses that may occur in the wires may also have caused the galvanic effect [44,45].

### 3.2. Statistical Analysis Results

In the pH 7 Ringer’s solution, the current density of the Gold retainer group was found to be significantly higher than the other groups (*p* < 0.05). The current density of the 3-braided SS and Dead Soft retainer groups were found to be statistically higher than the 6-braided SS, Titanium Grade 1, and Titanium Grade 5 retainer groups (*p* < 0.05). The corrosion rate of the Dead Soft and the 3-braided SS retainer groups were found to be significantly higher than the 6-braided SS, Titanium Grade 1, Titanium Grade 5, and Gold retainer groups (*p* < 0.05). The corrosion rate of the Gold retainer group was found to be statistically higher than the 6-braided SS, Titanium Grade 1, and Titanium Grade 5 retainer groups (*p* < 0.05). The polarization resistance of the Titanium Grade 5 retainer group was found to be significantly higher than the other retainer groups (*p* < 0.05). The polarization resistance of the Titanium Grade 1 retainer group was found to be statistically higher than the 3-braided SS, 6-braided SS, Gold, and Dead Soft retainer groups (*p* < 0.05). The polarization resistance of the 6-braided SS retainer was found to be significantly higher than the 3-braided SS, Gold, and Dead Soft retainer groups (*p* < 0.05). The polarization resistance of the 3-braided SS and Dead Soft retainer groups were found to be statistically higher than that of the Gold retainer group (*p* < 0.05).

In the pH 3.5 Ringer’s solution, the current density of the Gold retainer group was found to be significantly higher than the other retainer groups (*p* < 0.05). The current density of the Dead Soft retainer group was found to be statistically higher than the 6-braided SS, Titanium Grade 1, and Titanium Grade 5 retainer groups (*p* < 0.05). The current density of the 3-braided SS and 6-braided SS retainer groups were found to be significantly higher than the Titanium Grade 1 and Titanium Grade 5 retainer groups (*p* < 0.05). The current density of the Titanium Grade 1 retainer group was found to be statistically higher than the Titanium Grade 5 retainer group (*p* < 0.05). The corrosion rate of the Dead Soft retainer was found to be significantly higher than the other retainer groups (*p* < 0.05). The corrosion rate of the 3-braided SS retainer, Gold and 6-braided SS retainer groups were found to be statistically higher than Titanium Grade 1 and Titanium Grade 5 retainer groups (*p* < 0.05). The polarization resistance of the Titanium Grade 5 retainer group was found to be significantly higher than the other retainer groups (*p* < 0.05). The polarization resistance of the Titanium Grade 1 and the 6-braided SS retainer groups were found to be statistically higher than the 3-braided SS, Gold, and Dead Soft retainer groups (*p* < 0.05). The polarization resistance of the Dead Soft retainer group was found to be significantly higher than the 3-braided SS and Gold retainer groups (*p* < 0.05).

In the results of the comparison between the Ringer’s solutions, there was no statistical difference between the pH 7 Ringer solution and pH 3.5 Ringer solution in terms of the current density, corrosion rate, and polarization resistance of the 3-braided SS, Titanium Grade 1, and Gold retainer groups (*p* > 0.05). The current density and corrosion rate of the 6-braided SS and Dead Soft retainer groups were found to be significantly higher in the pH 3.5 Ringer solution than in the pH 7 Ringer solution (*p* < 0.05). There was no statistical difference between the pH 7 and pH 3.5 Ringer’s solutions in terms of their polarization resistance (*p* > 0.05). While there was no statistical difference between the pH 7 Ringer solution and the pH 3.5 Ringer solution in terms of current density and corrosion rate in the Titanium Grade 5 group, the polarization resistance was found to be statistically significantly higher in the pH 7 Ringer solution than in the pH 3.5 Ringer solution (*p* < 0.05).

### 3.3. Results of Scanning Electron Microscopy (SEM) Studies on Samples

**Figure 6:**
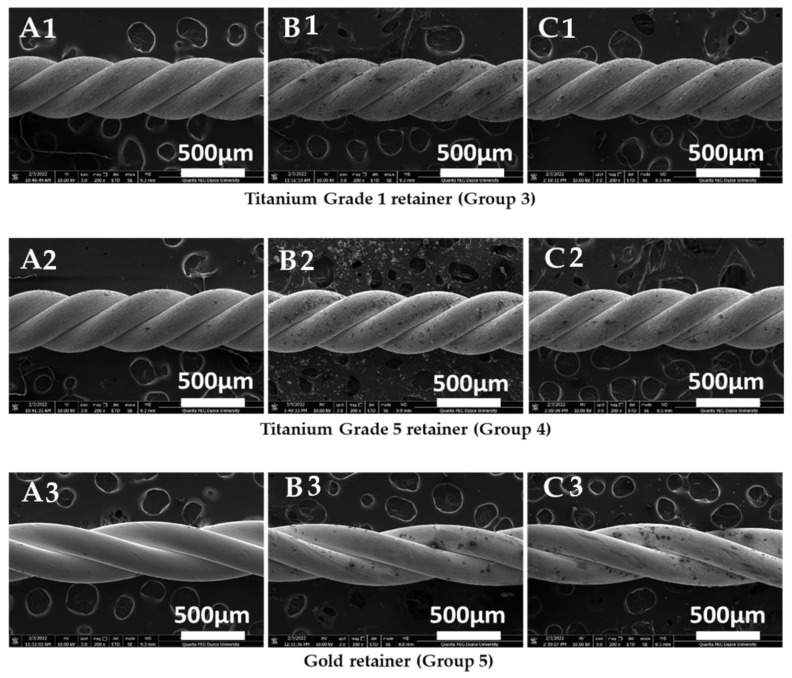
Scanning electron microscopy observations at 200× magnification. For Group 3 (**A1**); Before Experiment, (**B1**); pH 7 Ringer’s solution, (**C1**); pH 3.5 Ringer’s solution. For Group 4 (**A2**); Before Experiment, (**B2**); pH 7 Ringer’s solution, (**C2**); pH 3.5 Ringer’s solution. For Group 5 (**A3**); Before Experiment, (**B3**); pH 7 Ringer’s solution, (**C3**); pH 3.5 Ringer’s solution. (Scale bars for all groups: HV 10.00 kV, spot 3.0, mag 200×, det ETD, mode SE, WD 8.3–9.8 mm, 500µm, Quanta FEG).

After the electrochemical corrosion tests were performed, a sample was taken from each group, and images were obtained in a scanning electron microscope at 200× magnification (Figure 5, Figure 6). Pitting corrosion was observed on the 3-braided SS, 6-braided SS, and Dead Soft retainer groups in both solutions (Figure 5). No physical corrosion damage was observed on the Titanium Grade 1, Titanium Grade 5, and Gold retainer groups in both solutions (Figure 6).

## 4. Discussion

In previous studies, a favorable environment for the deterioration of dental material has been reported in the oral cavity because of temperature changes, changing pH, tooth brushing, chewing, dental plaque, ingested foods, moisture, and the presence of microorganisms [44,46,47,48,49]. In addition, Castro et al. reported that corrosion is an electrochemical process that leads to metal degradation [8]. Huang and Lin et al. have stated that the stainless steel used in orthodontic treatment increased its resistance to corrosion by forming a Cr_2_O_3_/Fe_2_O_3_ layer, and nickel-titanium by forming a TiO_2_ layer. This layer is defined as the passive oxide layer [14,50].

Pakshir et al. stated that the current density of stainless steel archwires (G&H Wire Company, Greenwood, India) was higher than nickel-titanium archwires (Orthotechnology Co. Ltd., Tampa, Florida). It was stated that the current density is directly proportional to the corrosion rate; a great current density shows lower resistance against corrosion, and the corrosion rate of nickel-titanium archwire was found to be lower than stainless steel [32]. Barcelos et al. stated that the current density and corrosion rate of stainless steel (Morelli Orthodontiaa, Rio de Janeiro, Brazil) archwires were lower than nickel-titanium (Morelli Orthodontiaa, Rio de Janeiro, Brazil) archwires. It has also been stated that stainless steel wire is less susceptible to corrosion, and that the current density and corrosion rate increase as the pH decreases [34]. Malkiewicz et al. stated that the lowest current density was in nickel-titanium archwires (RMO, USA: 3M, USA), while the highest current density was in stainless steel archwires (RMO, USA: 3M, USA). The current density of stainless steel archwires was found to be statistically higher than titanium-molybdenum and nickel-titanium archwires. The current density of titanium-molybdenum archwires was found to be statistically higher than nickel-titanium archwires [39].

In the present study, it was found that the 3-braided SS and Dead Soft retainer groups in the pH 7 Ringer solution had a statistically higher current density and higher corrosion rate than the Titanium Grade 1, Titanium Grade 5, and 6-braided SS retainer groups. It was found that the 3-braided SS, 6-braided SS and Dead Soft retainer groups in the Ringer’s solution with pH 3.5 had a significantly higher current density and higher corrosion rate than the Titanium Grade 1 and Titanium Grade 5 groups. The current density of the Titanium Grade 1 retainer group was found to be statistically higher than the Titanium Grade 5 retainer group in the Ringer’s solution with a pH of 3.5. This can be explained by the fact that the Titanium Grade 5 group consists of Ti-6Al-4V. Due to the aluminum and vanadium in Ti-6Al-4V, it is more resistant to corrosion than other types of titanium [51]. The current density of the Gold retainer group was significantly higher than the other retainer groups in both solutions. However, the corrosion rate of the Gold retainer group was significantly higher than the Titanium Grade 1 and Titanium Grade 5 retainer groups in both solutions. The equivalent weight and density of the gold affected the corrosion rate. The deterioration rate may change with the change in the material content. Noble metals, such as gold and platinum, are normally stable [52]. However, in the present study, it was observed that the Gold retainer group was corroded, and it is thought that the elements in the Gold retainer group may cause this situation by forming galvanic couples [53]. The present study’s demonstration of the higher corrosion resistance of titanium-containing wires was promoted by the study of Pakshir et al. [32] and Malkiewicz et al. [39]. It could not be promoted by the study of Barcelos et al. [34]. The data obtained from the present study and the studies in the literature show that orthodontic wires are corroded. Due to the methodological differences, it is not possible to directly compare the studies; however, this condition was stated in the study of Malkiewicz et al. [39].

In the study conducted by Huang with artificial saliva with pH 2.5, 3.5, 5.0, and 6.25, it was stated that the current density increased as the pH decreased [54]. Wajahat et al. stated in the study on nickel titanium wires (Ortho Organizer, USA) that the corrosion rate increased as the pH decreased; hence, the corrosive effect of acidic solutions is higher [29].

In the present study, the current density and corrosion rate of the 3-braided SS, Titanium Grade 1, Titanium Grade 5, and Gold retainer groups did not show any significant difference in the Ringer solution with pH 3.5 and pH 7. The current density and corrosion rate of the 6-braided SS and Dead Soft retainer groups were found to be statistically significantly higher in the Ringer solution with pH 3.5 than in the Ringer solution with pH 7. The present study was promoted by the studies of Huang [54] and Wajahat et al. [29].

Ziebowicz et al. stated that the polarization resistance of the NiTi archwire (American Orthodontics, Sheboygan, WI, USA) was higher than that of the CuNiTi archwire (Ormco Corporation, Brea, CA, USA) [16]. Lin et al. stated, in their study of acidic artificial saliva using linear polarization curve, that the Rp values were between 10^3^–10^4^ Ω.cm^2^, and there was a statistical difference between the polarization resistance of the different stainless steel bracket brands (3M Unitek, Puchheim, Germany: Dentaurum, Pforzheim, Germany: Ormco, Scafati, Italy: RMO, Denver, Col.: Tomy, Tokyo, Japan). However, there was no statistical difference between the bracket types (Roth type and Standard type) [50].

In the present study, the Rp values of the stainless-steel retainer groups were between 10^4^ and 10^5^ Ω.cm^2^ for the different pH levels. In both solutions, the polarization resistance of the Titanium Grade 1 and Titanium Grade 5 retainer groups was found to be statistically higher than the other groups. This can be explained by the high corrosion resistance of titanium-containing materials [51]. The polarization resistance of the Titanium Grade 5 group was found to be statistically higher than the Titanium Grade 1 group. This can be explained by the Ti-6Al-4V content of the Titanium Grade 5 group. Due to the aluminum and vanadium in Ti-6Al-4V, it is more resistant to corrosion than other types of titanium [51]. The polarization resistance of the 6-braided SS retainer was found to be significantly higher than the 3-braided SS, Dead Soft, and Gold retainer groups. The least polarization resistance was obtained in the Gold retainer group.

Lee et al. stated, in their study in artificial saliva solution with 0.01%, 0.1%, 0.25%, and 0.5% NaF concentrations using linear polarization curves, that the polarization resistance of nickel-titanium archwires decreased with the increase in the fluorine content, and the resistance against corrosion decreased [55].

In the present study, the polarization resistance of the 3-braided SS, 6-braided SS, Titanium Grade 1, Gold, and Dead Soft retainer groups showed no statistically significant difference between the pH 3.5 and pH 7 Ringer’s solution. The polarization resistance of the Titanium Grade 5 retainer group was found to be statistically higher in the pH 7 Ringer solution than in the pH 3.5. Ringer solution.

Li et al. stated that pitting corrosion occurs in nickel-titanium archwires (Shenzhen Superline Technology Co. Ltd., Guangdong, China) [56]. Kao and Huang stated, in the study in pH 4 artificial saliva solution, that stainless steel and nickel-titanium archwires’ (3M, Unitek, Monrovia, CA, USA) pitting corrosion was noted. They stated that acidic environments cause the wire to become fragile, and nickel-titanium wires can break under stress [57]. Suarez et al. stated that manufacturing errors are frequent in SS archwires (Ormco Corp., Glendora, CA, USA) and the surface structure is quite distorted after polarization tests. They stated that NiTi, CuNiTi, and TMA (Ormco Corp., Glendora, CA, USA) archwires have high resistance to corrosion with minimal structural damage [58]. Wajahat et al. stated that pitting corrosion occurred on nickel-titanium archwires [29].

In the present study, pitting corrosion occurred on the 3-braided SS, 6-braided SS, and Dead Soft retainer groups, while pitting corrosion did not occur on the Titanium Grade 1, Titanium Grade 5, and Gold retainer groups. While the corrosion resistance of the Gold retainer group was lower than Titanium Grade 1 and Titanium Grade 5, pitting corrosion was not observed on the Gold retainer group in the SEM images.

## 5. Conclusions

The current density of the Gold retainer group was found to be statistically higher than the other retainer groups in both solutions, indicating that its resistance to corrosion is less than the other groups. The corrosion rate of the Dead Soft retainer group was found to be statistically higher than the other retainer groups in both solutions, indicating that its corrosion resistance was lower than the other groups. The polarization resistance of the Titanium Grade 5 retainer group was found to be statistically higher than the other retainer groups in both solutions, indicating that its corrosion resistance was higher than the other groups. While pitting corrosion was not observed in the SEM images of the Titanium Grade 1, Titanium Grade 5, and Gold retainer groups, pitting corrosion was observed in the 3-braided SS, 6-braided SS, and Dead Soft retainer groups. Due to the retainer wires staying in the mouth for a long time, and as a result of electrochemical corrosion tests and SEM images, the use of titanium-containing retainer wires can be recommended in retention due to their high resistance to corrosion. Considering that the study was performed in vitro using a Ringer’s solution, further studies should be conducted in in vitro and in vivo environments that simulate the oral conditions.

## Figures and Tables

**Table 1:** Percentage of elements in assessed retainers for calculating equivalent weight (EW) and theoretical density (TD).

	Fe (%)	Cr (%)	Ti (%)	Ni (%)	Ag (%)	Cu (%)	Pt (%)	Al (%)	V (%)	Au (%)	EW(g)	TD (g/cm^3^)
Group 1	74	18	0	8	0	0	0	0	0	0	27.688	7.81
Group 2	73	18	0	9	0	0	0	0	0	0	27.702	7.82
Group 3	0	0	100	0	0	0	0	0	0	0	11.97	4.5
Group 4	0	0	90	0	0	0	0	6	4	0	11.720	4.43
Group 5	0	0	0	0	16	9	13	0	0	62	10.363	17.23
Group 6	74	18	0	8	0	0	0	0	0	0	27.688	7.81

EW: Equivalent weight, TD: Theoretical density, Fe: Iron, Cr: Crom, Ti: Titanium, Ni: Nickel, Ag: Siver, Cu: Copper, Pt: Platinum, Al: Aluminum V: Vanadium, Au: Gold.

**Table 2:** Current density (icor(µA/cm^2^)) values.

	*I_cor_* (µA/cm^2^)		
Groups (n = 5)	pH 7 Ringer’s Solution (Mean ± sd)	pH 3.5 Ringer’s Solution (Mean ± sd)	*p*-Value
Group 1	1.04 ± 0.68	1.05 ± 0.59	0.917 ^m^
Group 2	0.27 ± 0.19	0.66 ± 0.24	* 0.047 ^m^
Group 3	0.22 ± 0.09	0.25 ± 0.09	0.917 ^m^
Group 4	0.12 ± 0.02	0.13 ± 0.04	0.251 ^m^
Group 5	2.43 ± 0.86	4.34 ± 2.89	0.117 ^m^
Group 6	1.01 ± 0.13	1.78 ± 0.63	* 0.047 ^m^
*p*-value	0.000 ^K^	0.000 ^K^	

K: Kruskal-Wallis test, m: Mann-Whitney U test, n: Number of samples, *: *p* < 0.05, *p*: Significance value, sd: Standard deviation, *I_cor_* (µA/cm^2^): Current density.

**Table 3:** Corrosion rate (mm/year) values.

	Corrosion Rate (mm/year)		
Groups (n = 5)	pH 7 Ringer’s Solution (Mean ± sd)	pH 3.5 Ringer’s Solution (Mean ± sd)	*p*-Value
Group 1	0.012 ± 0.008	0.012 ± 0.007	0.917 ^m^
Group 2	0.003 ± 0.002	0.008 ± 0.003	* 0.047 ^m^
Group 3	0.002 ± 0.001	0.002 ± 0.001	0.917 ^m^
Group 4	0.001 ± 0.000	0.001 ± 0.000	0.251 ^m^
Group 5	0.005 ± 0.002	0.009 ± 0.006	0.117 ^m^
Group 6	0.012 ± 0.002	0.021 ± 0.007	* 0.047 ^m^
*p*-value	0.001 ^K^	0.000 ^K^	

K: Kruskal-Wallis test, m: Mann-Whitney U test, n: Number of samples, *: *p* < 0.05, *p*: Significance value, sd: Standard deviation.

**Table 4:** Polarization resistance (*R_p_* (Ωcm^2^)) values.

	*R_p_* (Ωcm^2^) × 10^4^		
Groups (n = 5)	pH 7 Ringer’s Solution (Mean ± sd)	pH 3.5 Ringer’s Solution (Mean ± sd)	*p*-Value
Group 1	7.28 ± 6.45	3.48 ± 1.91	0.175 ^m^
Group 2	21.06 ± 12.61	11.29 ± 6.07	0.076 ^m^
Group 3	34.66 ± 25.90	18.62 ± 4.05	0.175 ^m^
Group 4	47.32 ± 7.45	34.12 ± 10.07	* 0.047 ^m^
Group 5	2.40 ± 0.75	1.82 ± 1.22	0.117 ^m^
Group 6	5.00 ± 0.50	5.77 ± 3.81	0.602 ^m^
*p*-value	0.000 ^K^	0.000 ^K^	

K: Kruskal-Wallis test, m: Mann-Whitney U test, n: Number of samples, *: *p* < 0.05, *p*: Significance value, sd: Standard deviation.

## Data Availability

All of the data supporting the results of this study are included within the article.
